# Alterations in intestinal microbiota in ultramarathon runners

**DOI:** 10.1038/s41598-022-10791-y

**Published:** 2022-04-28

**Authors:** Mika Sato, Yoshio Suzuki

**Affiliations:** Juntendo University Graduate School of Health and Sports Science, 1-1, Hiragagakuendai, Inzai, Chiba 270-15 Japan

**Keywords:** Clinical microbiology, Microbiome, Predictive markers

## Abstract

To date, only one study has reported changes in the gut microbiome of an ultramarathon runner before and after competing in the race. Herein we aimed to investigate changes in intestinal microbiota in nine ultramarathon runners. Eight of the nine participants ran 96.102 km (up 8062 m, down 6983 km) and one ran 99.12 km (up 8448 m, down 7369 m) within 38–44 h. Intestinal microbiota alterations were examined at three timepoints: before (Pre), after (Post), and 10 days after (Recovery) the race. The α- and β-diversity of intestinal microbiota were unaffected by the race. Six of the nine participants showed the B-type enterotype, while the remaining three showed the P-type enterotype; however, significant difference between enterotypes was not observed in the influence of the ultramarathon on intestinal microbiota. The abundance of mean *Faecalibacterium prausnitzii*, was significantly decreased from 2.9% (Pre) to 1.6% (Post), as well as other three butyrate producing bacteria. One participant with the largest decrease in *F. prausnitzii* abundance (− 85.7%) reported sluggishness and shallow sleep from Post to Recovery. Our findings revealed that the abundance of butyrate-producing bacteria is decreased in ultramarathon runners, which consequently decreases butyrate levels in the intestine and affects host immune function.

## Introduction

The human intestinal microbiota is composed of at least approximately 1000 species and 100 trillion microorganisms, mostly dominated by bacteria. There exists an intimate symbiosis between humans and intestinal microbiota, and pertinent bacterial species perform a vast array of functions, such as digestion, vitamin production, immune stimulation, pathogen growth inhibition, energy source production, and detoxification. Moreover, they are involved in immune function, consequently playing a pivotal role in maintaining host health and physiological function^1^. Intestinal microbiota is influenced by not only exercise habits^2,3^ but also exercise programs^4–6^. Several potential mechanisms, involving gut-associated lymphoid tissue, exercise-induced heat stress, ischemia, and changes in barrier function, have been suggested via which exercise alters intestinal microbiota^7^.

The effects of a single marathon on intestinal microbiota have been previously reported in half-^8^ and full-marathon^9^ runners. However, only one such ultramarathon-based study has been reported; briefly, Grosicki et al*.* examined changes in the gut microbiome of an ultramarathon runner before and after competing in the Western States Endurance Run (WSER), a 163-km mountain footrace, reporting most rapid and pronounced shifts in gut microbiome composition after acute exercise^10^. The increase in *Veillonella* abundance in intestinal microbiota has been observed in after both full marathon^9^ and WSER^10^. However, *Veillonella* is known to be associated with P-type enterotype which has high *Prevotella* abundance^11^. In contrast, the Japanese predominantly show B-type enterotype which has high *Bacteroides* abundance^11–13^. In addition, the influence of single stage ultramarathon with longer duration has not been examined. Therefore, we hypothesized that the influence of ultramarathon with longer duration gave the different influence on gut microbiota of Japanese runners from the previous studies^8–10^; especially, it is notable that ultramarathon-induced changes in B-type enterotype have not been reported to date.

In this study, we investigated changes in intestinal microbiota in participants competing in the Trans Japan Alps Race 2020 (TJAR2020), which is a major 415-km single-stage ultramarathon that takes place in the Japanese Alps Mountains. TJAR2020 was cancelled once started due to stormy weather caused by a typhoon. We herein examined runners who had reached > 61.5 km (up 6035 m; down 3145 m) when the decision was made to cancel the race 31 h after the start and who had arrived at Shinhotaka Onsen within 38–44 h of starting the race.

## Results

Nine runners (ID: A to I) participated in this study. Their characteristics (mean ± SD) before the race were as follows: age = 46.9 ± 5.8 years, height = 171.1 ± 6.1 cm, weight = 63.3 ± 4.6 kg, body water = 41.2 ± 3.3 L, protein = 11.1 ± 0.9 kg, minerals = 3.8 ± 0.3 kg, body fat = 7.2 ± 1.7 kg, muscle mass = 53.0 ± 4.3 kg, lean body mass = 56. 1 ± 4.5 kg, skeletal muscle mass = 31.7 ± 2.6 kg, BMI = 21.6 ± 1.1 kg/m^2^, and body fat = 11.4% ± 2.7%.

The race started from the coast of Toyama Bay (altitude 0 m). The participants had crossed Tsurugi-dake (altitude 2999 m, 36.858 km from the start) and Yakushi-toge (altitude 2294 m, 61.5 km from the start) when the race was cancelled 31 h after the start and had reached Shinhotaka Onsen (altitude 1117 m, 96.102 km from the start) within 38–44 h of starting the race (Supplementary Figure S1). Eight of the nine participants ran 96.102 km (up 8062 m; down 6983 km) and one participant (ID: A) ran 99.12 km (up 8448 m; down 7369 m) (Supplementary Figure S2).

During the race, the intake values were as follows: energy = 3831.6 ± 710.3 kcal, protein = 84.2 ± 21.4 g, fat = 147.6 ± 64.6 g, carbohydrate = 545.7 ± 103.9 g, and sodium = 13.3 ± 8.8 g (salt equivalent). Further, the energy expenditure and physical activity levels of participants during the race were 13,789 ± 943 kcal (331 ± 24 kcal/h) and 4.89 ± 0.16, and the energy balance was − 9,957 ± 517 kcal.

For analyses, we collected spontaneously excreted feces before (Pre), after (Post), and 10 days after the race (Recovery). Intestinal microbiota was analyzed by 16S rRNA-based large-scale genome sequencing, yielding raw data of 26,564 ± 2683 reads. Subsequently, 19,689 ± 621 reads were filtered, and 18,255 ± 765 reads passed the filter. The 10,000 reads randomely selected from that passed through the filter were used for operational taxonomic unit (OTU) analysis. This eventually led to the identification of 2,828 OTUs, 9 phyla (Supplementary Figure S3), 156 genera (Supplementary Figure S4), and 380 species.

In case of Pre-samples, hierarchical clustering of intestinal microbiota at the genus level revealed that six participants showed B-type enterotype with a high proportion of *Bacteroides* and the remaining three (ID: B, F, and G) showed P-type enterotype with a high proportion of *Prevotella* (Supplementary Figure S5). In addition, the *Firmicutes*/*Bacteroides* ratio was higher in case of B- than for P-type enterotypes at Pre; however, no significant change was observed between Pre and Post nor between Pre and Recovery (Supplementary Figure S6). Linear discriminant analysis effect size (LEfSe, p-value cut off = 0.1 FDR adjusted) did not find a significantly different bacteria between B- and P-types at all levels from phylum to OTU in both at Pre and Post (data not shown). Comparison of abundance (Pre vs Post) at the species levels showed eight and one significantly changed bacteria in B- and P-types, respectively, while no bacteria showed significant difference in both B- and P-types (Supplementary Table S1). Therefore, in subsequent comparisons at the OTU and species levels, all nine participants were analyzed as a single group without considering differences in enterotypes.

**Figure 1:**
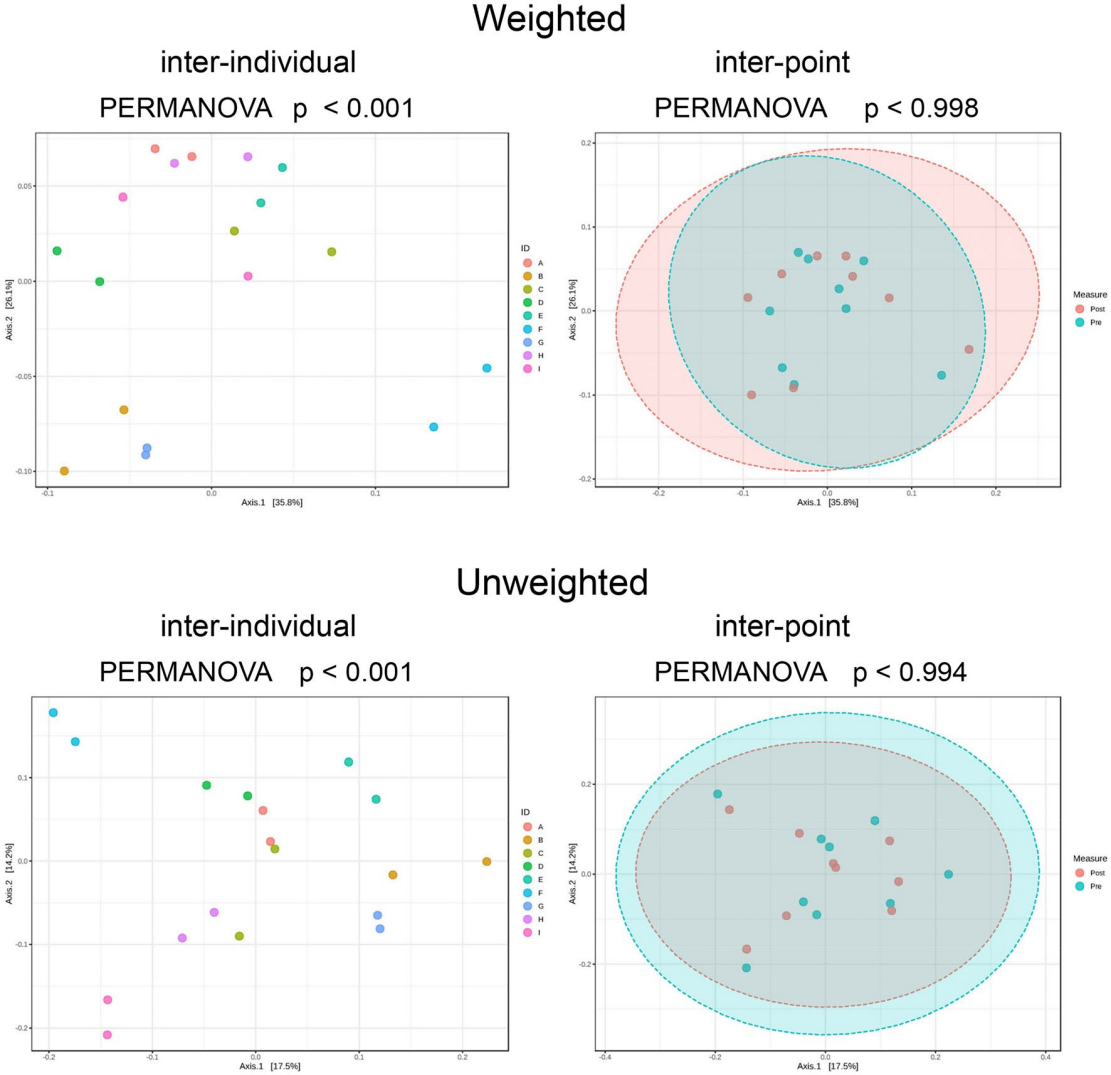
Comparison of β-diversity. Significant interindividual differences were observed in both weighted (p < 0.001) and unweighted (p < 0.001) PCoA, whereas no differences were observed before (Pre) and after (Post) the race.

No significant changes in α-diversity indices (OTU level; ACE, Chao1, Fisher, Observed, Shannon, and Simpson) were found between Pre and Post (Table 1). Besides, β-diversity comparisons (OTU level) showed significant interindividual differences in both weighted (p < 0.001) and unweighted (p < 0.001) principal coordinate analysis (PCoA), but no differences were detected between Pre and Post (Fig. 1).

**Table 1:** Comparison of α-diversity indices of intestinal microbiota (OTU level) before (Pre) and after (Post) the race.

	Pre		Post		p
	Mean	SD	Mean	SD	
ACE	483.8	157.2	445.6	141.5	0.269
Chao1	504.7	148.6	446.6	143.9	0.118
Fisher	59.0	21.1	57.4	16.1	0.681
Observed	299.6	83.5	294.4	63.7	0.734
Shannon	3.695	0.247	3.789	0.316	0.174
Simpson	0.938	0.035	0.938	0.052	0.986

**Figure 2:**
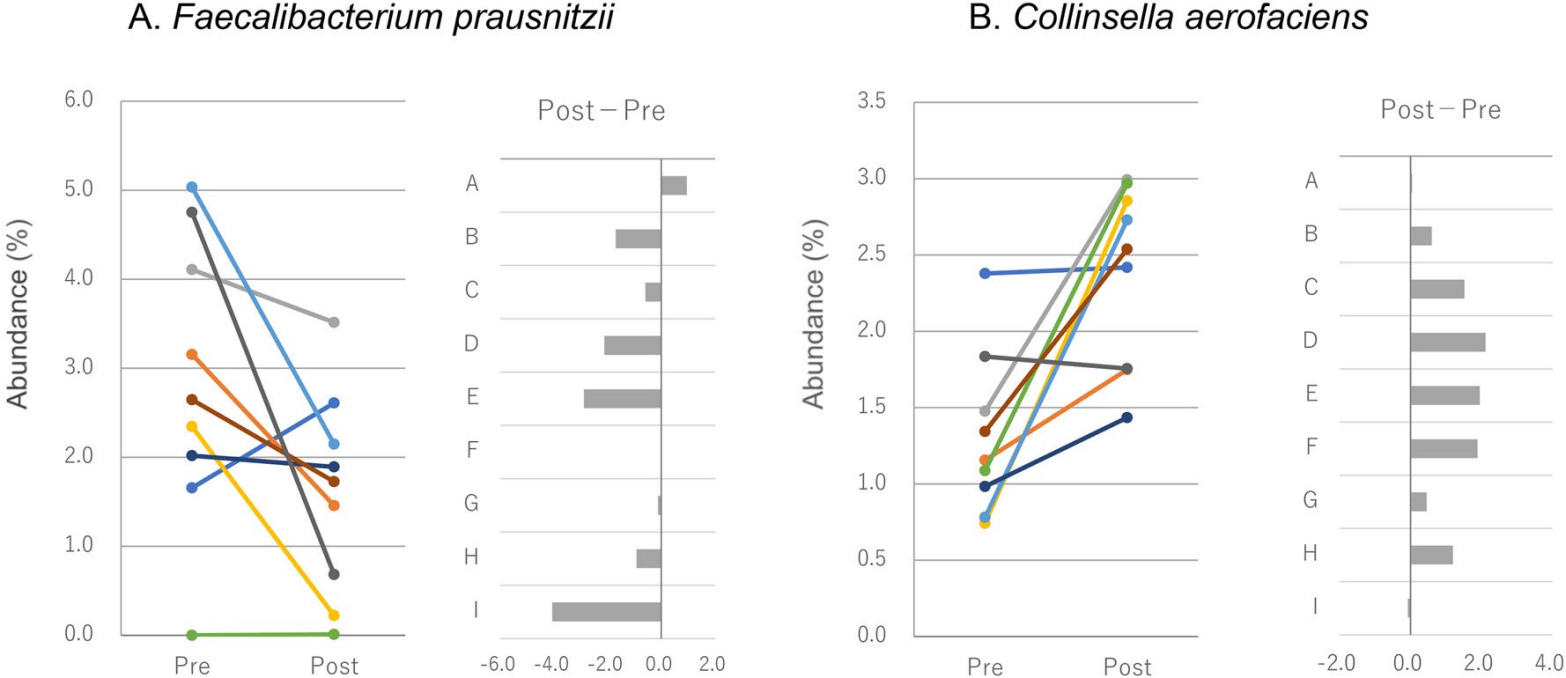
Changes in abundance (%) of *Faecalibacterium prausnitzii* (**A**) and *Collinsella aerofaciens* (**B**) before (Pre) and after (Post) the race.

LEfSe (p-value cut off = 0.1 FDR adjusted) did not find significantly different bacteria between Pre and Post at all levels from phylum to OTU (data not shown). Since LEfSe does not support paired data, we then tried to find bacteria that changed before (Pre) and after (Post) the race. The Wilcoxon signed rank sum test was applied to compare the abundance of bacterial species between Pre and Post. A significant increase was found in the abundance of *Collinsella aerofaciens*, *Catenibacillus scindens*, *Clostridium* sp E2, *Ruminococcaceae* bacterium LM158, *Alistipes putredinis*, *Drancourtella massiliensis*, *Massiliomicrobiota timonensis*, *Clostridium* sp Culture Jar 17, *Romboutsia timonensis*, and *Streptococcus sanguinis*, and a significant decrease was noted in the abundance of *Blautia luti, Eubacterium rectale, Faecalibacterium prausnitzii,* and butyrate-producing bacterium SS3/4 (Table 2). *F. prausnitzii* and *C. aerofaciens* showed a change of > 1% (mean value) in their abundance between Pre and Post. The abundance of *F. prausnitzii* decreased in seven and that of *C. aerofaciens* increased in eight of the nine participants (Fig. 2).

**Table 2:** Bacterial species with a significant change in abundance before (Pre) and after (Post) the race.

Name	Pre (%)		Post (%)		Change	p
	Mean	SD	Mean	SD		
*Collinsella aerofaciens*	1.3	0.5	2.4	0.6	Up	0.005
*Catenibacillus scindens*	< 0.1	< 0.1	< 0.1	< 0.1	Up	0.014
*Clostridium* sp E2	< 0.1	< 0.1	< 0.1	< 0.1	Up	0.014
*Ruminococcaceae* bacterium LM158	0.1	0.2	0.4	0.5	Up	0.014
*Alistipes putredinis*	0.1	0.2	0.2	0.2	Up	0.022
*Blautia luti*	< 0.1	< 0.1	< 0.1	< 0.1	Down	0.032
*Drancourtella massiliensis*	0.1	0.2	0.2	0.2	Up	0.035
*Massiliomicrobiota timonensis*	< 0.1	< 0.1	0.1	0.1	Up	0.036
*Clostridium* sp Culture Jar 17	< 0.1	< 0.1	0.1	0.1	Up	0.036
*Eubacterium rectale*	< 0.1	< 0.1	< 0.1	< 0.1	Down	0.036
*Romboutsia timonensis*	< 0.1	0.1	0.4	0.7	Up	0.039
*Faecalibacterium prausnitzii*	2.9	1.6	1.6	1.1	Down	0.041
Butyrate-producing bacterium SS3/4	0.3	0.3	0.2	0.3	Down	0.042
*Streptococcus sanguinis*	< 0.1	< 0.1	< 0.1	< 0.1	Up	0.042

*Veillonella atypica* was the only bacteria belonging to the genus *Veillonella*. Scheiman et al*.* reported an increase in *Veillonella* relative abundance in runners postmarathon, and *V. atypica* was isolated from stool samples^9^. However, in this study, *V. atypica* abundance at Post was < 0.1% in all but one participant, and no significant changes were present between Pre and Post.

On assessing the relationship between diet and intestinal microbiota, we found a negative correlation between changes in *Ruminococcaceae* bacterium LM158 abundance (Post to Pre) and intake of lipids (g/kg BW, ρ =  − 0.750, p = 0. 020), energy (kcal/kg BW, ρ =  − 0.733, p = 0.025), and sodium (salt equivalent, g/kg BW, ρ =  − 0.733, p = 0.025) during the race (Supplementary Figure S7A–C). In contrast, a positive correlation was found between changes in *D. massiliensis* abundance (Post to Pre) and intake of protein (g/kg BW, ρ = 0.812, p = 0.008) (Supplementary Figure S7D).

The α-diversity indices (OTU level) did not show any significant differences between Recovery and Pre [ACE (p = 0.359), Chao1 (p = 0.133), Fisher (p = 0.496), Observed (p = 0.426), Shannon (p = 0.673), and Simpson (p = 0.652)]. In addition, in terms of β-diversity (OTU level), no significant differences were seen in both weighted (p < 1) and unweighted (p < 0.997) PCoA on comparing Pre and Recovery, whereas significant interindividual differences were detected in both weighted (p < 0.001) and unweighted (p < 0.001) PCoA.

On comparing Pre and Recovery using the Wilcoxon signed rank sum test, we found a significant decrease in the abundance of only four species: *B. luti* (p = 0.020), *Clostridium glycyrrhizinilyticum* (p = 0.020), *Bacteroides dorei* (p = 0.027), and *Sutterella wadsworthensis* (p = 0.036).

One participant (ID: I) reported sluggishness and shallow sleep from Post to Recovery. In this participant, *F. prausnitzii* abundance was 4.75% at Pre, which was the second highest among all participants, but it decreased to 0.68% at Post (− 85.7%), which was the seventh highest among all participants (Fig. 2), and a slight increase to 1.34% was noted at Recovery. None of the participants reported any changes in physical conditions from Recovery to the following 2 weeks.

## Discussion

In the study conducted by Grosicki et al*.*, the Shannon index (α-diversity) of intestinal microbiota in case of a runner who participated in a 163-km ultramarathon was 2.78 ± 0.05 between 21 weeks before and 10 days after the race^10^. This variation was observed in only one runner, and although an increase or decrease was detected depending on the time of the measurement, the change was small^10^. The Shannon index (α-diversity) of intestinal microbiota in case of our study participants was 3.695 ± 0.247 at Pre and 3.789 ± 0.316 at Post, with no significant changes observed between Pre and Post. It is notable that β-diversity was not affected by race, although significant differences were found among individuals. Collectively, our findings suggest that intestinal microbiota diversity is more likely to vary among individuals than to be affected by ultramarathon.

In the case report by Grosicki et al*.*, abundance of *Faecalibacterium* genera after an ultramarathon (approx. 20%) decreased from Pre-Race (approx. 25%)^10^. Although the abundance of *F. prausinizii* was namely reported, the post-race decrease of *Faecalibacterium* seems consistent with this study.

A decrease in *F. prausnitzii* abundance is reportedly associated with various diseases^14–20^. The anti-inflammatory effects of *F. prausnitzii* are apparently mediated by its products, such as butyrate^21,22^. Furthermore, *F. prausnitzii* abundance is evidently associated with peripheral T-cell subsets^23^. In this study, we observed a decrease in *F. prausnitzii* abundance as well as three other butyrate producing bacteria. As importance of butyrate has been reported to systemic immunity^24,25^, the decrease in the butyrate producing bacteria could affect the immunity of ultramarathon runners.

Ultramarathon^26–31^ runners often experience upper respiratory tract infections after the race; acute exercise has been suggested to increase the risk of infection (open window hypothesis) via immune suppression^32–35^. However, the effects of a single bout of exercise on immune function is a controversial topic. For example, dense population at competitions can itself increase the risk of infection^36,37^, and immunity is not only affected by exercise but also sleep and nutrition^38^. Our study participants did not report any symptoms (e.g., fever, cough, runny nose, and sore throat) of upper respiratory tract infections from Post to Recovery and even up to 2 weeks after Recovery. However, one participant showing the highest decrease in *F. prausnitzii* abundance reported sluggishness and shallow sleep from Post to Recovery. It is notable that the number of participants in TJAR2020 was limited to 30; further, all athletes were required to take certain measures for infection prevention, such as wearing a mask until the start of the race. Therefore, the risk of infection owing to dense population was negligible. In addition, as TJAR2020 is a single-stage ultramarathon, it is necessary to sleep and eat during the race. Considering these facts, the ultramarathon, including sleep and nutrition, could affect immune function of the participant through the decrease in butyrate-producing bacteria such as *F. prausnitzii* abundance.

*C. aerofaciens* is widely distributed in the human intestine. A study found its prevalence to be lower in patients with irritable bowel syndrome than in healthy individuals^39^, while another study reported contradictory results^40^. Moreover, *C. aerofaciens* is reportedly an obesity biomarker^41^. Despite this, its influence on host physiology remains to be explained. Similar to a previous study that observed an increase in its abundance in half-marathon runners^8^, even in this study, *C. aerofaciens* abundance showed an increase in seven of the nine participants after the race (p = 0.005). Therefore, we believe that endurance running can increase the abundance of *C. aerofaciens*. However, the race duration of half-marathon and TJAR2020 is considerably different, and accordingly, energy expenditure is markedly different too. Further studies are thus warranted to elucidate the relationship between *C. aerofaciens* abundance and endurance running.

Scheiman et al*.* reported a significant increase in *Veillonella* relative abundance in runners postmarathon^9^. As *Veillonella* produces propionate from lactate, a model was proposed in which *Veillonella* converts lactate produced during exercise to propionate in the intestinal tract, contributing to improved performance^9^. An increase in the abundance of *Veillonella* was also detected after the 163-km WSER^10^. In contrast, in this study, only *V. atypica* was detected from the genus *Veillonella*. Its abundance was < 0.1% in all participants, except in one participant at Post, and no significant changes were present between Pre and Post. The energy expenditure during the WSER is typically 13,000–16,000 kcal^42^. The runner (age 32 y) reported by Grosicki et al. finished the WSER 2019 in the top-10^10^; his finish time should be about 16 h according to the race record (https://www.wser.org/results/2019-results/, accessed on April 16, 2021). Thereby, his energy expenditure was estimated to be ≥ 800–1000 kcal/h. In contrast, the energy expenditure in case of our study participants was 294–372 kcal/h. As evident, in comparison with full marathon and the WSER, the energy expenditure per hour was lesser, and the contribution of the glycolytic system was thereby considered to be smaller. We believe that less lactate production could be responsible for the differences observed between our results and those previously reported.

The changes observed in intestinal microbiota in this study were inconsistent with those observed in half-^8^ and full marathon runners^9^. A single-stage ultramarathon lasts long enough to require eating and sleeping during the race^43^, and this seems to have impacted our results. Our findings suggest that changes in intestinal microbiota in ultramarathon runners need to be considered separately from those in half- or full marathon runners.

*Ruminococcaceae* bacterium LM158^44^ and *D. massiliensis*^45^ abundance was found to show a significant change before and after a race, and the extent of change was correlated with energy and nutrient intake during the race. Although genomic information is available for both species, no information exists pertaining to nutrient requirements. The number of the participants was limited to nine, and the correlation seems to be dependent on data related to three (*Ruminococcaceae* bacterium LM158) or two (*D. massiliensis*) participants. Therefore, further studies need to be conducted to verify the validity of such correlations.

Intestinal microbiota rapidly responds to environmental changes: dietary changes cause significant variations within a day, with recovery occurring within 2 days of returning to a normal diet^46^. Herein intestinal microbiota diversity did not differ between Pre and Recovery, and only four of the 380 species identified showed differences in their abundance. Thereby, intestinal microbiota was considered to have recovered to the Pre-race condition at Recovery.

As the race was cancelled 31 h after the start because of the inclement weather, the runners had to hurry descend by the nearest route and return home on their own. The announcement of the cancellation was emailed to the individual runners, but since they were in the middle of the race, the timing of when the individual runners learned of the cancellation varied. Some of them arrived at Shinhotaka Onsen in pieces between 38 and 44 h after the start. We caught the descending runners at Shinhotaka Onsen and asked individual runners to cooperate with our study. It was in stormy weather, in late evening to night, and the runners were so disappointed at the race cancellation and even more exhausted by the race that we could not require them many things. This situation gave some limitation for this study. The timing of the stool collection should be important. We asked to collect the first spontaneously excreted stool after the arrival at Shinhotaka Onsen, assuming the race-route was from start to Shinhotaka Onsen. However, Shinhotaka Onsen was not originally the goal, the runners arrived there 7–13 h after the official cancellation, and the stool excreted during the descent, if any, could not be collected. Therefore, it was not possible to control the timing of post-race stool collection.

In addition to the major limitation described above, this study has a few limitations. As TJAR2020 is a single-stage ultramarathon, participants are required to eat and sleep during the race. Both sleep^47^ and diet potentially affect intestinal microbiota. In this study, we investigated the effects of diet, but not sleep. To understand ultramarathon-induced changes more comprehensively, it is pivotal to assess the relationship between sleep and intestinal microbiota too. Antibiotic use is known to potently influence microbiome composition, although we did not check the use. This study did not measure the change in metabolites that could help elucidate the impact of the ultramarathon on gut microbiota. Regarding the physical condition of participants, an interview was conducted approximately 1 month after the race, which may have led to recall bias. The effects of the race on immune function should have been comprehended, for example, by maintaining a logbook or examination by a physician. Finally, the number of the participants was limited to nine; further studies involving more participants are warranted to validate the accuracy of our results.

To summarize, we herein examined changes in intestinal microbiota before and after participation in ultramarathon, with the aim of assessing the influence of ultramarathon on intestinal microbiota in the Japanese. A decrease in the abundance of butyrate-producing bacteria, such as *F. prausnitzii,* was observed after the race, indicative of a decrease in butyrate levels in the intestine, which is bound to have affected the immune function of our study participants. Further, *C. aerofaciens* abundance was found to increase after the race. In addition, ultramarathon-induced changes in intestinal microbiota were independent of the enterotype. Further studies are warranted to elucidate the influence of diet and sleep on intestinal microbiota and also to assess the effects on host immune function.

## Methods

### Participants

We herein included nine male runners (aged 40–56 years old) who had participated in TJAR2020. All of them had completed a full marathon within 3 h and 20 min or a 100-km marathon within 10 h and 30 min within 2 years of TJAR2020.

The inclusion criteria were as follows: (1) participant of TJAR2020 (scheduled on Aug 8, 2021) and (2) good physical condition and ability to safely participate in this study. The exclusion criteria were as follows: (1) suffering from or having a history of serious cardiovascular, hepatic, renal, respiratory, endocrine, or metabolic disorders, (2) suffering from or having a history of hepatitis B or C, (3) having had 200 mL blood drawn within 1 month or 400 mL blood drawn within 3 months prior to TJAR2020, and (4) having participated in a clinical trial within 3 months prior to TJAR2020 or being a current participant of a clinical trial.

Eligible runners were informed in writing as well as orally of the purpose, methods, and expected results of the study, the protection of personal information and disclosure of study results, and the possibility of withdrawing their consent to participate. They understood the information shared with them and voluntarily signed a consent form to participate in this study.

This study was approved by the ethical review committee of Juntendo University Graduate School (no. 2021-17 and 2021-89 for intestinal microbiota and 2021-26 for dietary survey and body measurements). This study was conducted in accordance with the ethical standards of the 1964 Declaration of Helsinki and its later amendments or comparable ethical standards.

### Study design

Naturally excreted feces were obtained from all participants at three timepoints: Pre (Aug 5–7), Post (first feces after the race), and Recovery (Aug 18–20). As gut microbiota has substantial intra-individual temporal variability that exceeds the inter-individual variability^48^ e.g. rapid change with diet^46^, control is needed to confirm intra-individual variability. A case report by Grosicki et al. showed an apparent change in gut microbiota after an ultramarathon, 2 h after the race, compared to baseline (21 weeks before the race), Pre-Race (2 weeks before the race) and Recovery (10 days after the race)^10^. With reference to this case-report, this study designed to use Pre-race (within 3 days before the race) as baseline and Recovery (10 days after the race) to confirm the return to the baseline.

The participants measured their weight/body composition using InBody 470 (InBody Japan, Tokyo, Japan), and Pre-race weight with ware and luggage to be carried on their back was measured at the reception site of Mirage Land (Uozu City, Toyama, Japan) approximately 8 h before the start of the race.

The race started from the coast of Toyama Bay (altitude 0 m) at 00:00 on Aug 8, 2021. The participants had crossed Tsurugi-dake (altitude 2999 m, 36.858 km from the start) and Yakushi-toge (altitude 2294 m, 61.5 km from the start) when the race was cancelled 31 h after the start and had reached Shinhotaka Onsen (altitude 1117 m, 96.102 km from the start) within 38–44 h of starting the race (Supplementary Figure S1).

The participants were interviewed to assess their physical condition from the goal of the race until the collection of feces at Recovery and for 2 weeks thereafter. The reports submitted by the participants to the TJAR2020 office were obtained from the office.

### Energy expenditure

Eight participants ran 96.102 km (up 8062 m, down 6,983 m), and one participant ran 99.12 km (up 8448 m, down 7,369 m) (Supplementary Figure S2). The distance and cumulative altitude difference were calculated by the geographic information system software Kashmir 3D v9.3.7 (DAN Sugimoto, http://www.kashmir3d.com/, accessed on Aug. 7, 2021).

Energy consumption during the race was calculated using this formula, which was previously used to estimate energy expenditure during mountaineering by Nakahara et al*.*^49^:

Energy expenditure (kcal) = [1.8 × duration (h) + 0.3 × distance (km) + 10.0 × cumulative altitude of up (km) + 0.6 × cumulative altitude of down (km)] × [body weight (kg) + luggage to be carried (kg)].

The body and luggage weights were measured before the race and used for [body weight (kg) + luggage to be carried (kg)].Physical activity levels during the race were calculated using energy expenditure and basal metabolic standard values listed in the Dietary Reference Intakes for Japanese (2020).

### Dietary intake

The intake of energy, protein, fat, carbohydrate, and sodium (salt equivalent) during the race was calculated based on food intake reported by the participants to the TJAR2020 office and nutrition facts label on each food item.

### Energy balance

Energy balance was calculated by subtracting energy expenditure from energy intake during the race.

### Intestinal microbiota

All participants placed the stool collection sheet “Raku-Ryu Cup” (Takahashi Katasei, Tokyo, Japan) on the toilet seat, followed by collecting their stool sample and sampling an aliquot (c.a. 0.3 g) into a tube containing zirconia beads (BioMedica Science, Tokyo, Japan) and 2 mL RNA Later^®^ (Thermo Fisher Scientific, Tokyo). The lid of the tube was then tightly closed, and the mixture was vigorously shaken approximately 40 times. The stool samples were subsequently stored in a refrigerator (4 °C) until needed for DNA extraction.

To the stool samples (150 µL) with RNA Later®, 850 µL TE buffer (10 mM Tris–HCl, 10 mM EDTA) containing RNase A (final concentration 100 µg/mL; Invitrogen, Japan) and lysozyme (final concentration 3.0 mg/mL; Sigma, Tokyo, Japan) was added, followed by incubation for 1 h at 37 °C with gentle shaking. Subsequently, purified achromopeptidase (final concentration 2,000 U/mL; Fuji Film, Tokyo, Japan) was added, and the mixture was further incubated at 37 °C for 30 min. Then, sodium dodecyl sulfate (final concentration 1%) and proteinase K (final concentration 1 mg/mL; Nacalai, Tokyo, Japan) were added, followed by incubation at 55 °C for 1 h.

DNA was extracted using phenol:chloroform:isoamyl alcohol (25:24:1), precipitated with isopropanol, washed with 75% ethanol, and dissolved in 200 µL TE buffer.

The V1-V2 region of the 16S rRNA gene was amplified by PCR using 27Fmod (5′-AGRGTTTGATYMTGGCTCAG $-$ 3′) and 338R (5′-TGCTGCCTCCCGTAGGAGT-3′) primers. The amplicons thus obtained (approximately 330 bp) were purified using AMPure XP (Beckman Coulter, Tokyo, Japan). The amount of DNA was then quantified using the Quant-iT™ Picogreen dsDNA Assay Kit (Invitrogen, Tokyo, Japan) on a TBS-380 Mini-Fluorometer (Turner Biosystems, Tokyo, Japan).

16S metagenomic sequencing was performed using MiSeq, according to the protocol recommended by Illumina. Two paired-end reads were merged by the fastq-join program based on overlapping sequences. Sequences with an average quality value of < 25 and inaccurate universal primers at both ends were excluded. The 10,000 reads from each sample that passed the quality filter were sorted in order of quality and classified into OTUs with a 97% pairwise-identity cutoff using the UCLUST program (Edgar 2010) v5.2.32 (https://www.drive5.com). Each OTU was classified against the GLSEARCH program and the genomic databases of RDP and National Center for Biotechnology Information.

DNA extraction, amplification, sequencing, and taxonomic assignment were conducted by MyMetagenome (Tokyo, Japan).

### Statistical analysis

Values represent mean ± SD. Intraindividual α-diversity and intestinal microbiota comparisons were achieved using the Wilcoxon signed rank sum test. PCoA was used for β-diversity comparisons (Pre vs. Post and Pre vs. Recovery).

The Spearman’s correlation coefficient (ρ) was used for correlation of the change in the bacterial abundance (Post to Pre) that showed significant changes in response to the race and intake of energy and nutrients during the race.

MicrobiomeAnalyst (https://www.microbiomeanalyst.ca/; accessed on Nov 10, 2021) and MetaboAnalyst 5.0 (https://www.metaboanalyst.ca/; accessed on Nov 10, 2021) were used for intestinal microbiota analyses. Correlation was analyzed by IBM SPSS v23 (IBM Japan, Tokyo, Japan). Statistical significance was set at p < 0.05.

## Supplementary Information

Supplementary Information.
