# Supplementary material for: Alterations in intestinal microbiota in ultramarathon runners
Source: Sci Rep. 2022 Apr 28;12:6984. doi: 10.1038/s41598-022-10791-y (PMC9050700; doi:10.1038/s41598-022-10791-y)
Supplement: Supplementary file 1 — Supplementary Information. [file 41598_2022_10791_MOESM1_ESM.pdf]

Alterations in intestinal microbiota in ultramarathon runners

Mika Sato, Yoshio Suzuki*

Juntendo University Graduate School of Health and Sports Science, 1-1, Hiragagakuendai, Inzai, Chiba 270-15, Japan

*Correspondence author: yssuzuki@juntendo.ac.jp

Table S1 Bacteria that showed significant change between Pre and Post stratified in enterotype

Name	B-type			P-type			Inconsistency in Down/Up
	Post/Pre	p.value		Post/Pre	p.value		
Catenibacillus_scindens	Down	0.03125	*	Down	0.37109		
Anaeromassilibacillus_senegalensis	Down	0.03125	*	Up	0.61798		Inconsistent
Romboutsia_timonensis	Down	0.03125	*	Down	0.92552		
Clostridium_sp__Culture_Jar_17	Down	0.03125	*	**Not detected**			
Collinsella_aerofaciens	Down	0.03308	*	Down	0.16543		
Streptococcus_gordonii	Down	0.03351	*	Down	0.52846		
Massiliomicrobiota_timonensis	Down	0.03602	*	Down	0.18351		
Ruminococcaceae_bacterium_LM158	Down	0.04005	*	Down	0.36303		
Clostridiaceae_bacterium_bSSV31	Down	1		Down	0.00035	*	

Paired comparison, non-parametric* $p < 0.05$

Anaeromassilibacillus senegalensis: Abundance increased in all six participants in B-type, while one increased but two decreased in P-type (not significant).

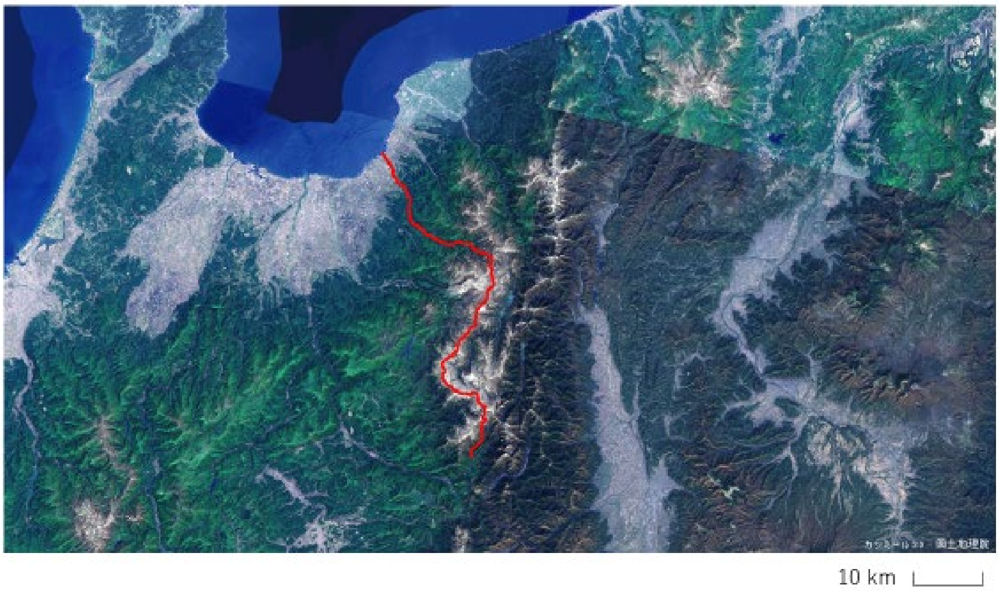

Figure S1 Ultramarathon racecourse: from Toyoma Bay to Shinhotata Onsen (red line)

A

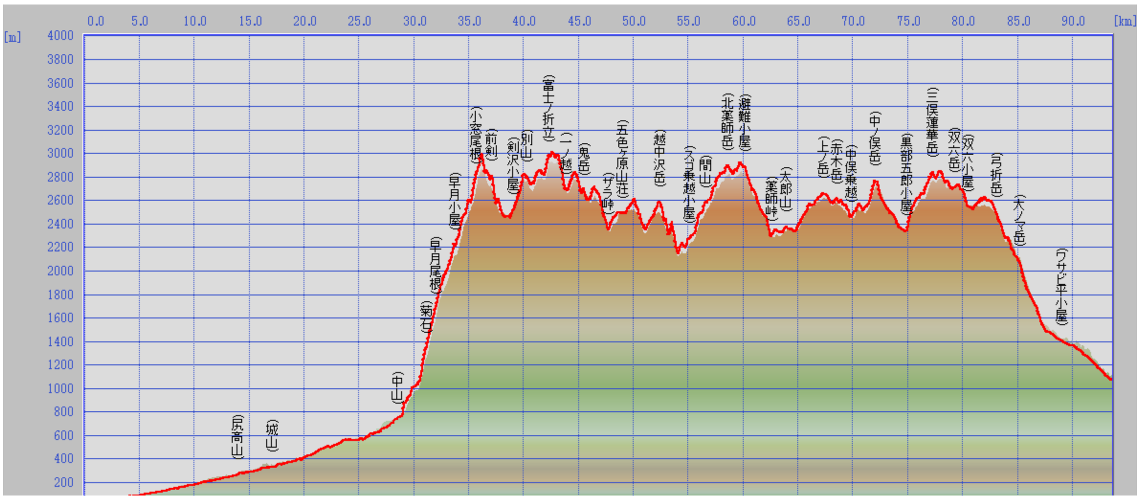

B

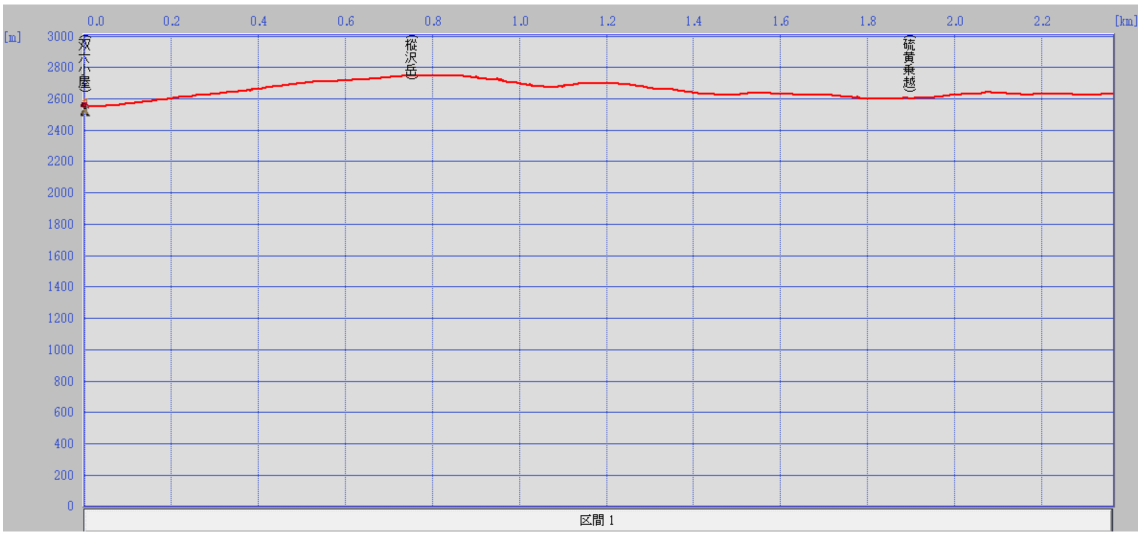

Figure S2 Cross-sectional view of the racecourse

Vertical axis shows elevation (m) and horizontal axis shows distance (km)

A. The course that eight participants (ID: B–I) ran

B.Additional course that one participant (ID: A) ran from Sugoroku-Goya (80.0 km from start) and back

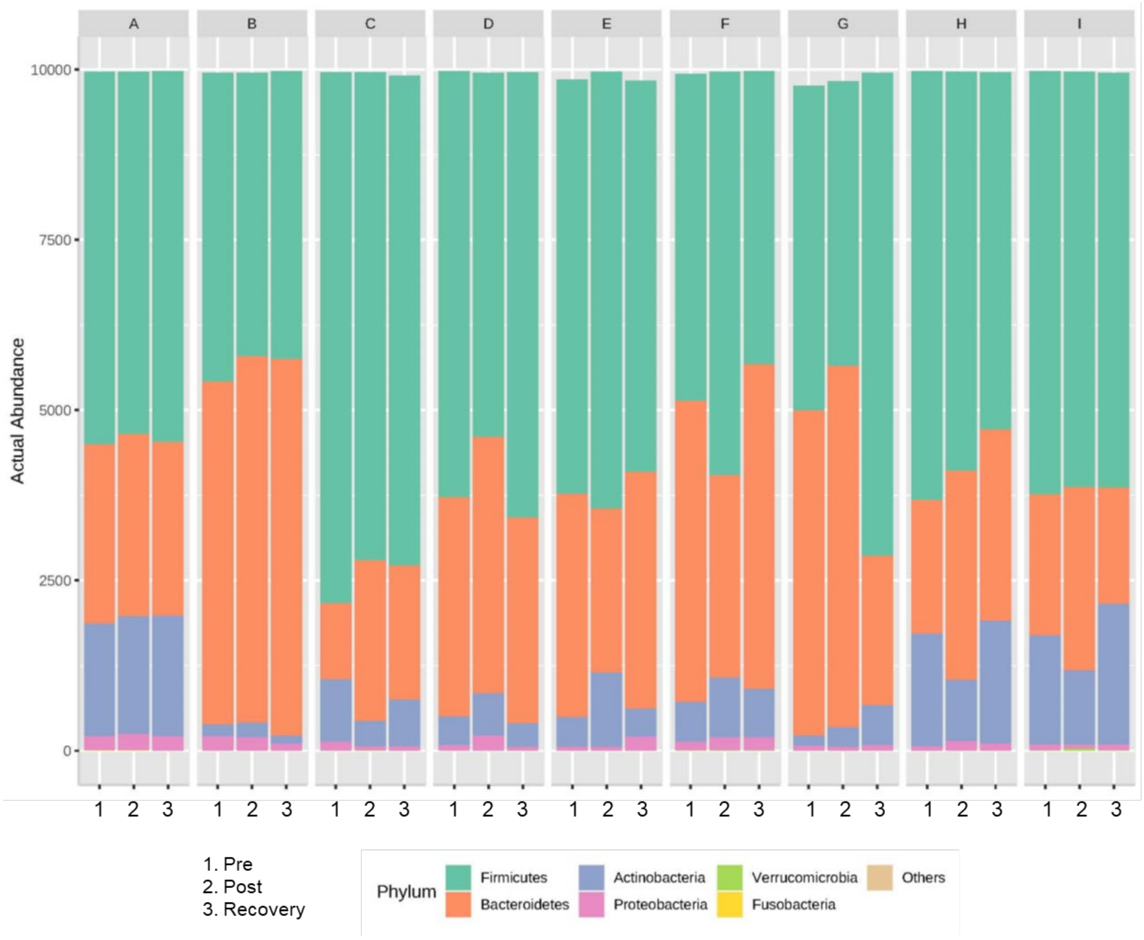

Figure S3 Relative abundance of intestinal microbiota at the phylum level

Alphabets (A–I) denote participants, and numbers (1–3) denote the sampling timepoint (Pre, Post, and Recovery). In phylum, others include *Spirochaetes*, *Synergistetes* and *Lentisphaerae*.

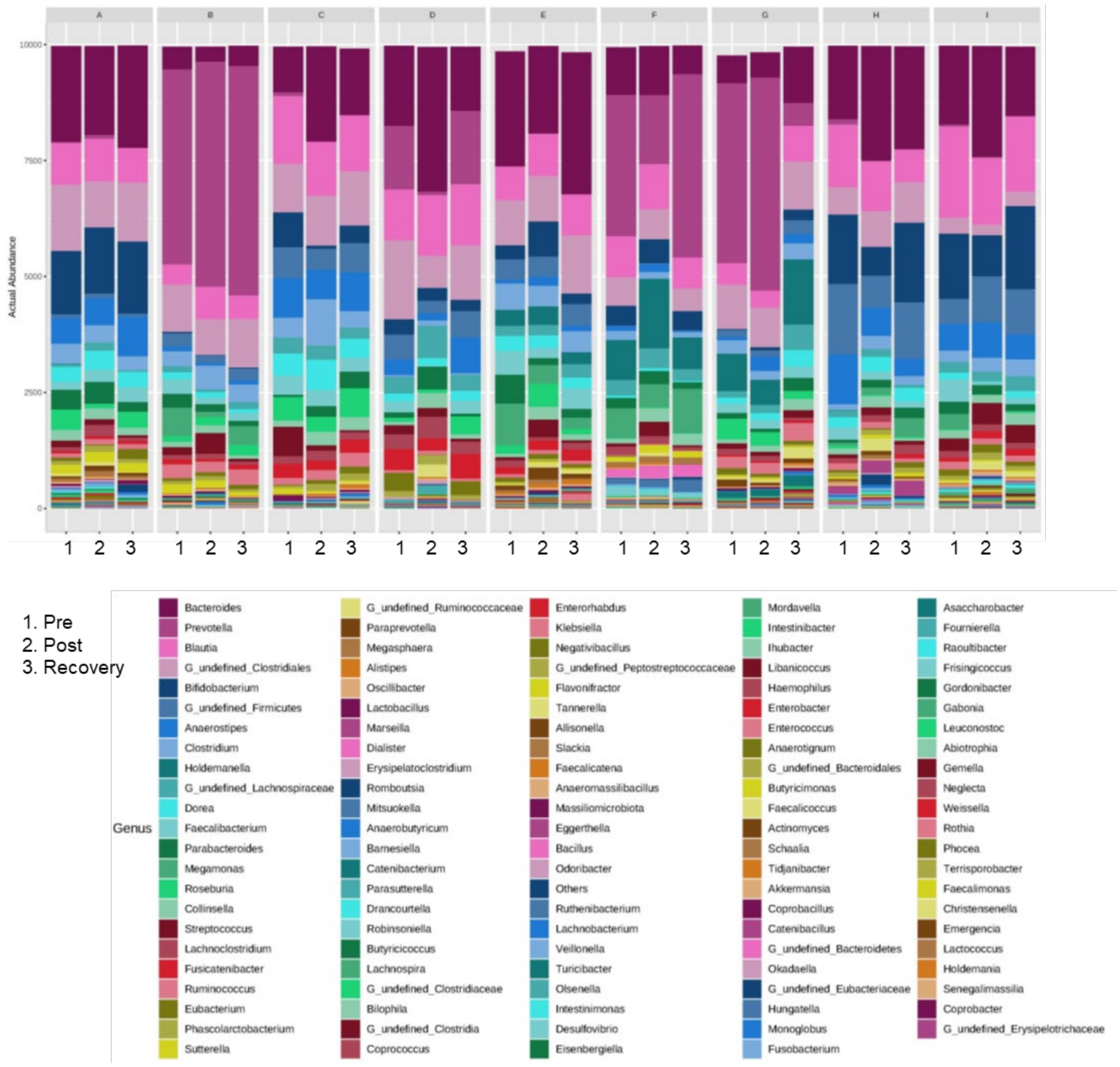

Figure S4 Relative abundance of intestinal microbiota at the genera level
Alphabets (A–I) denote participants, and numbers (1–3) denote the sampling timepoint (Pre, Post, and Recovery). Colors show 114 genera starting with the most abundance.

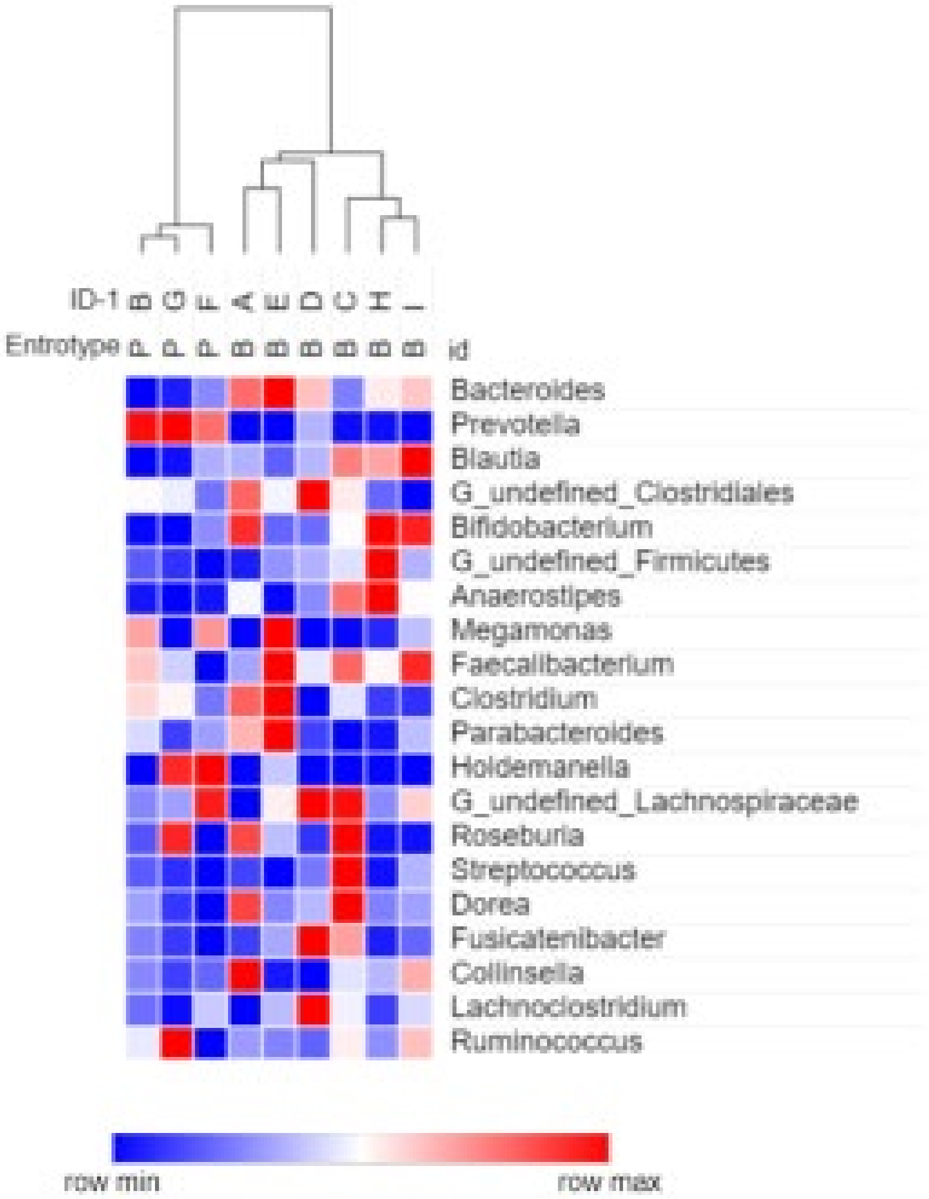

Figure S5 Hierarchical clustering of intestinal microbiota in at the genera level

The figure shows the genus level from most to 20. The results represent hierarchical clustering of all 156 genera (including “unidentified”). Six participants showed B-type enterotype and the remaining three showed P-type enterotype.

This figure was prepared using Morpheus, https://software.broadinstitute.org/morpheus.

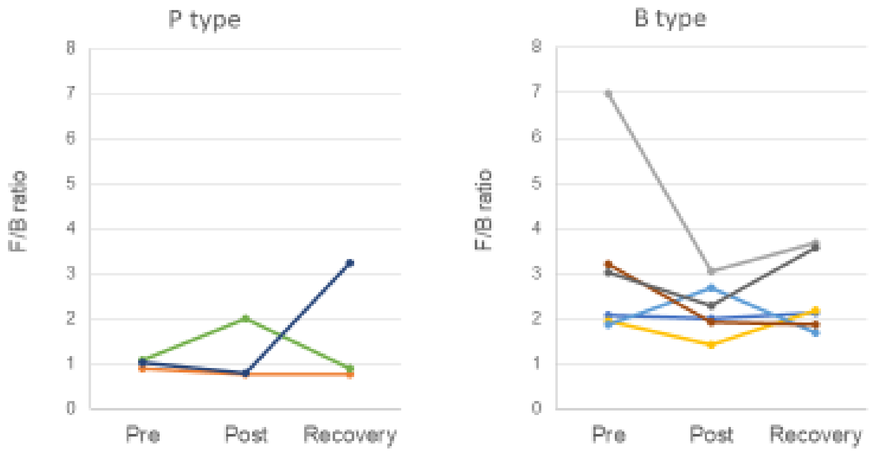

		Pre		Post		Recovery	
Entrotype	n	mean	SD	mean	SD	mean	SD
B type	6	3.2	1.9	2.2	0.6	2.5	0.9
P type	3	1.0	0.1	1.2	0.7	1.6	1.4
p		0.020 *		0.121		0.197	

Figure S6 *Firmicutes*/*Bacteroides* ratio (F/B ratio)

The F/B ratio was greater for B- than for P-type enterotype before the race (Pre), whereas there was no significant change between Pre and after the race (Post) or even between Pre and 10 days after the race (Recovery) for both enterotypes. *$p < 0.05$

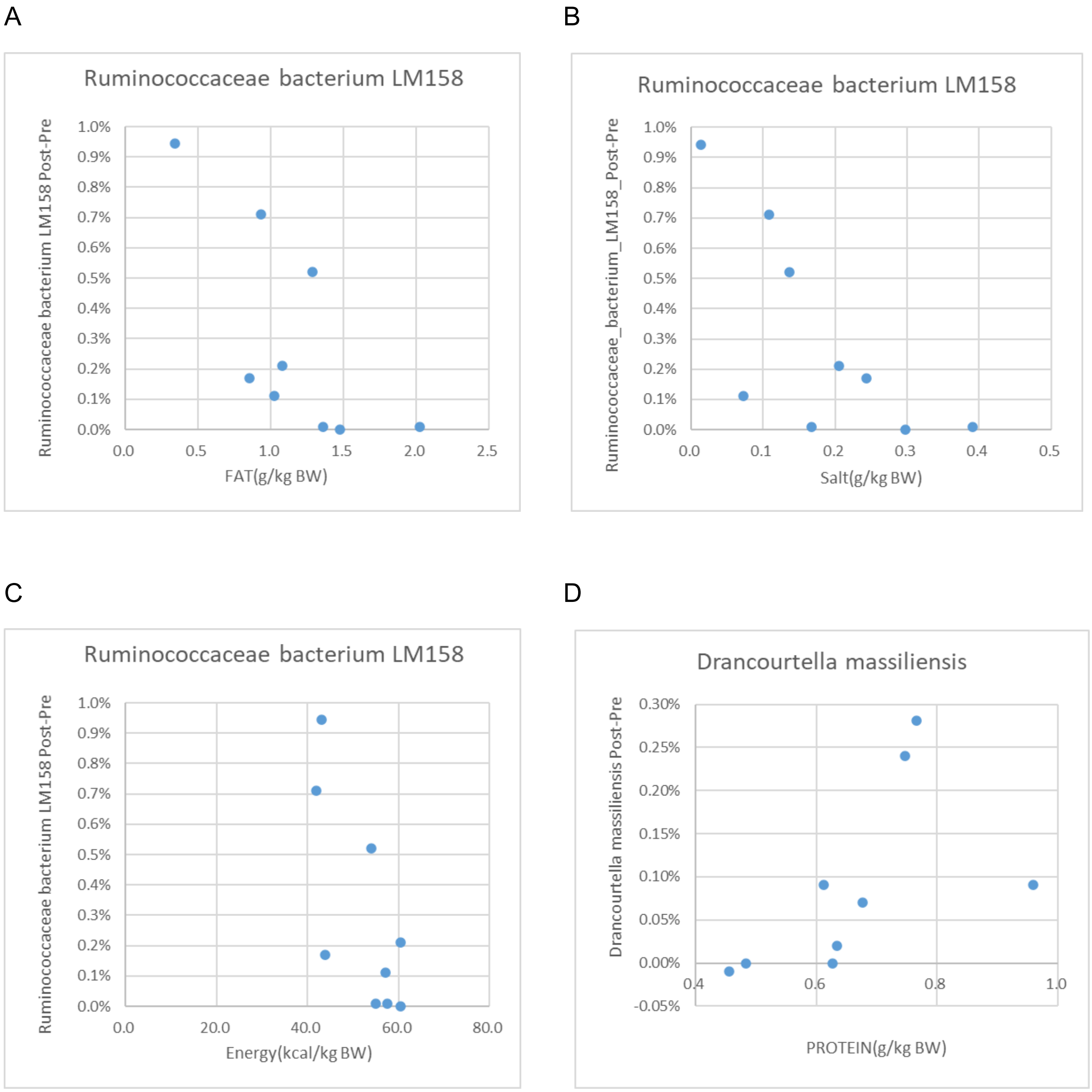

Figure S7 Scatter plot showing the relationship between dietary intake and changes in bacterial abundance (Post to Pre)

A. Fat intake (g/kg BW) vs. *Ruminococcaceae* bacterium LM158 (Post to Pre)
B. Salt intake (g/kg BW) vs. *Ruminococcaceae* bacterium LM158 (Post to Pre)
C. Energy intake (kcal/kg BW) vs. *Ruminococcaceae* bacterium LM158 (Post to Pre)
D. Protein intake (g/kg BW) vs. *Drancourtella massiliensis* (Post to Pre)

BW = body weight
